# WRH-2412 alleviates the progression of hepatocellular carcinoma through regulation of TGF-β/β-catenin/α-SMA pathway

**DOI:** 10.1080/14756366.2023.2185761

**Published:** 2023-03-13

**Authors:** Mohammed A. F. Elewa, Wagdy M. Eldehna, Ahmed M. E. Hamdan, Samraa H. Abd El-kawi, Asmaa M. El-Kalaawy, Taghreed A. Majrashi, Reham F. Barghash, Hatem A. Abdel-Aziz, Khalid S. Hashem, Mohammed M. H. Al-Gayyar

**Affiliations:** a Biochemistry Department, Faculty of Pharmacy, Kafrelsheikh University, Kafr El-Sheikh, Egypt; b Department of Pharmaceutical Chemistry, Faculty of Pharmacy, Kafrelsheikh University, Kafrelsheikh, Egypt; c Department of Medicinal Chemistry, Faculty of Pharmacy, King Salman International University (KSIU), South Sinai, Egypt; d Department of Pharmacy Practice, Faculty of Pharmacy, University of Tabuk, Tabuk, Saudi Arabia; e Department of Medical Histology and Cell Biology, Faculty of Medicine, Beni-Suef University, Beni-Suef, Egypt; f Department of Pharmacology, Faculty of Medicine, Beni-Suef University, Beni-Suef, Egypt; g Department of Pharmacognosy, College of Pharmacy, King Khalid University, Abha, Saudi Arabia; h Institute of Chemical Industries Research, National Research Centre, Dokki, Giza, Egypt; i Department of Applied Organic Chemistry, National Research Center, Dokki, Giza, Egypt; j Biochemistry Department, Faculty of Veterinary Medicine, Beni-Suef University, Beni-Suef, Egypt; k Department of Biochemistry, Faculty of Pharmacy, Mansoura University, Mansoura, Egypt; l Department of Pharmaceutical Chemistry, Faculty of Pharmacy, University of Tabuk, Tabuk, Saudi Arabia

**Keywords:** Alpha smooth muscle actin (α-SMA), E-cadherin, hepatocellular carcinoma, pyrazolo[34-*b*]pyridine, SMAD4, transforming growth factor (TGF)-β

## Abstract

Hepatocellular carcinoma is considered one of the most lethal cancers, which is characterised by increasing prevalence associated with high level of invasion and metastasis. The novel synthetic pyrazolo[3,4-*b*]pyridine compound, **WRH-2412**, was reported to exhibit *in vitro* antitumor activity. This study was conducted to evaluate the antitumor activity of **WRH-2412** in HCC induced in rats through affecting the TGF-β/β-catenin/α-SMA pathway. Antitumor activity of **WRH-2412** was evaluated by calculating the rat’s survival rate and by assessment of serum α-fetoprotein. Protein expression of TGF-β, β-catenin, E-cadherin, fascin and gene expression of SMAD4 and α-SMA were determined in hepatic tissue of rats. **WRH-2412** produced antitumor activity by significantly increasing the rats’ survival rate and decreasing serum α-fetoprotein. **WRH-2412** significantly reduced an HCC-induced increase in hepatic TGF-β, β-catenin, SMAD4, fascin and α-SMA expression. In addition, **WRH-2412** significantly increased hepatic E-cadherin expression.

## Graphical Abstract

**Figure:**
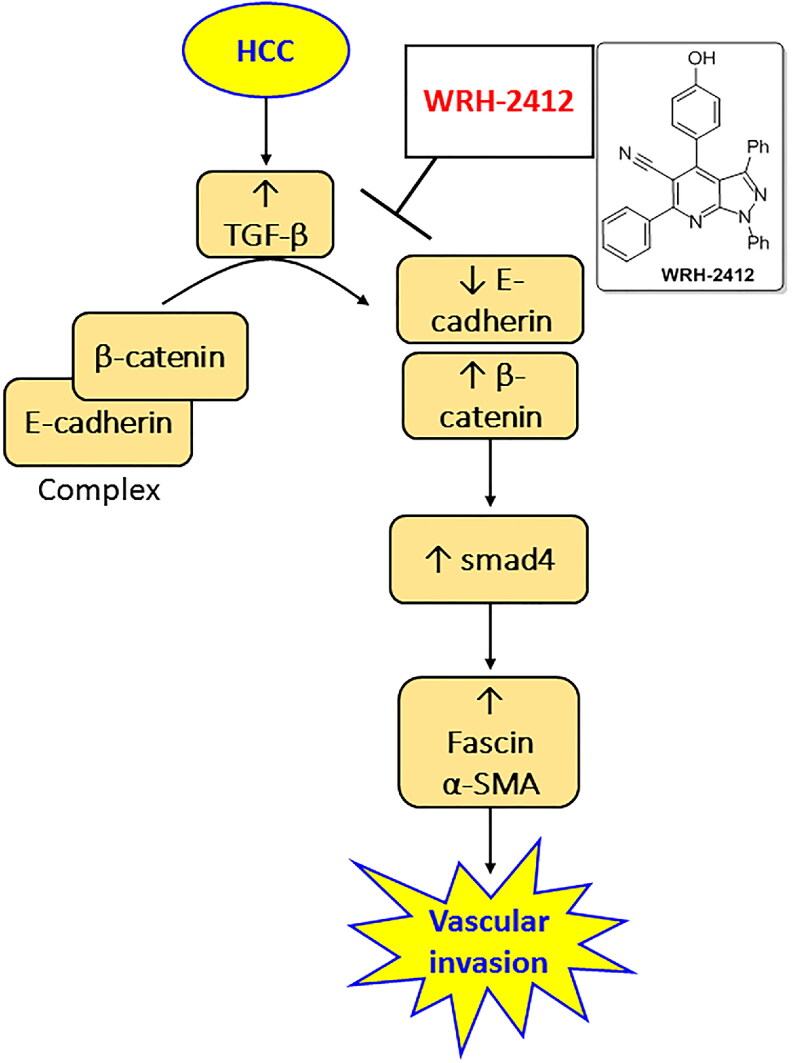

## Introduction

Hepatocellular carcinoma (HCC) is ranked as the sixth most frequent cancer in the world. It occupies the fourth leading cause of cancer-related death. Hepatocarcinogenesis process is considered as a complex multistep process that is affected by a group of risk factors leading to tumour progression and metastasis.^1^ It is characterised by several molecular modifications such as alterations in the gene expression of some growth factors, proteolytic enzymes, extracellular matrix components and inflammatory cytokines.^2^ Due to its rapid growth rate as well as the surrounding fibrotic tissue created by the chronic inflammation, HCC is featured as a highly hypoxic tumour.^2^ The median five-year survival of HCC patients is below 20% due to tumour recurrence and resistance to chemotherapy.^3^ Therefore, there is an urge to find new therapeutic agents to improve the overall survival in HCC patients.

**Figure 1.**
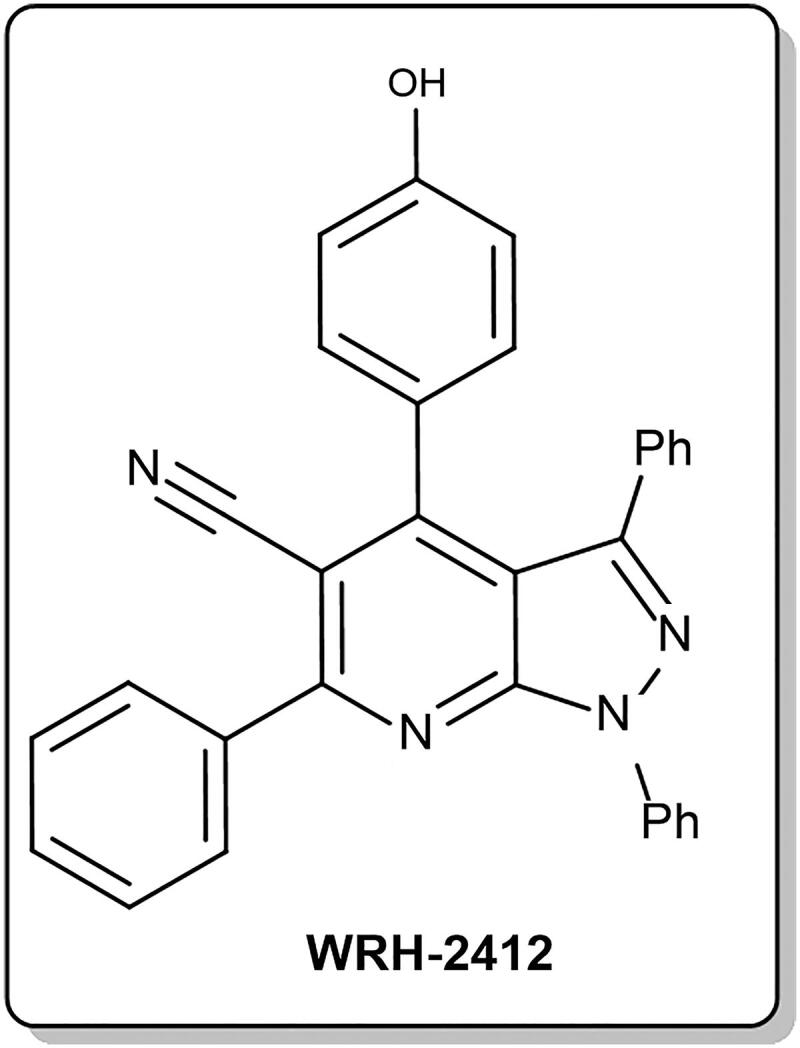
Chemical structure of **WRH-2412**.

One of the candidate therapeutic agents against HCC is pyrazolo[3,4-*b*] pyridine compounds, which are small molecules proved as antitumor,^4^^,^^5^ antibacterial,^6^ anti-inflammatory^7^ and antioxidant.^8^ Of these novel synthesised pyrazolo[3,4-*b*]pyridine compounds is 4–(4-hydroxyphenyl)-1,3,6-triphenyl-1*H*-pyrazolo[3,4-*b*]pyridine-5-carbonitrile, **WRH-2412** (Figure 1). Recently, **WRH-2412** has been proven to have promising *in vitro* anticancer action with broad spectrum anti-proliferative activities as disclosed through the US-NCI assays, with outstanding growth inhibition full panel GI_50_ (MG-MID) value equals 2.16 μM.^9^ Interestingly, the antitumor activity of **WRH-2412** against HCC hasn’t been investigated *in vitro* or *in vivo*.

TGF-β can regulate the different processes in HCC such as tumour proliferation, diffusion and metastasis through binding with both type I and II transmembrane receptor of the serine-threonine kinases leading to heterodimerization of SMAD3 with SMAD4.^10^ In addition, β-catenin is another important player that has a pivotal biological role in tumour development, growth and regeneration especially those of the liver ranging from hepatitis to HCC.^11^ Therefore, this study was conducted to evaluate the antitumor activity of **WRH-2412** in experimentally induced HCC in rats through affecting the TGF-β and β -catenin and their subsequent effect on the tumour invasion markers, fascin and α-SMA.

## Materials and methods

### Animal experiments

The animal protocol was approved by Institutional Animal Care and Use Committee, Beni-Suef University (BSU-IACUC), Approval number: 022–347. Forty Sprague–Dawley adult male rats weighing 180–200 g were used. All animals were kept under standard conditions of temperature (25 °C) with a regular 12 h light/12 h dark cycle. Each animal was examined daily and weighed weekly throughout the 16 weeks experiential period. They were classified randomly into five groups, each consisted of 10 rats.

- Control group: Rats recieved intraperitoneal (i.p.) injection of phosphate buffer saline (PBS, 10 mM, pH 7.4) twice weekly for 16 weeks.
- **WRH-2412**-treated control group: Rats were injected with 5 mg/kg i.p. **WRH-2412** twice weekly for 16 weeks.
- HCC group: Rats were i.p. injected with 200 mg/kg thioacetamide (TAA; Tocris Bioscience) twice weekly for 16 weeks.
- HCC treated with **WRH-2412**: Rats were i.p. injected with 5 mg/kg **WRH-2412** twice weekly for 16 weeks accompanied by 200 mg/kg TAA i.p., twice weekly for 16 weeks.

### Collection of rat samples

Blood samples were collected from retro-orbital plexus of each rat under brief thiopental sodium anaesthesia (40 mg/kg, i.p). Blood was centrifuged at 3000 rpm for 5 min and sera were separated and subsequently stored at −80 °C prior to further analysis. Whole rat livers were removed, dried, weighed, rinsed with normal saline and divided into two aliquots. One aliquot was fixed in 10% buffered formaldehyde for subsequent morphological analysis. The second portion was homogenised in 10 mM PBS, pH 7.4 and stored at −80 °C for biochemical analysis.

### Morphologic analysis of liver tissue

Liver samples were cut and fixed in 10% buffered formalin and embedded in paraffin wax. Five micrometer sections were cut and stained with hematoxylin and eosin (H&E). Liver sections were anonymously coded and examined in a blinded manner using a digital camera-aided computer system (Nikon Digital Camera, Japan).

### Immunohistochemistry

Immunohistochemical (IHC) analysis was performed on 5-μm paraffin sections cut from a paraffin block of liver. Sections were deparaffinized by heating at 55 °C for 1 h and then rehydrated using xylene and descending concentrations of ethanol. Sections were then subjected to microwave treatment in Antigen retrieval 6. All non-specific binding sites were blocked by using blocking buffer (10% FCS in 0.5% Triton-PBS). Sections were incubated with monoclonal antibodies for TGF-β and β-catenin (Abcam) in 1:500 dilution at 4 °C overnight. Then, were incubated with secondary antibodies (Envision + Dual Link System-HRP, Dakocytomation) conjugated to HRP. The used chromagen was 2% DAB in 50 mM Tris-buffer, pH 7.6. Slides were subsequently counterstained with (H&E).

### Evaluation of hepatoprotective effects

Hepatoprotective effects were assessed by measuring the serum activity of alanine aminotransferase (ALT) and aspartate aminotransferase (AST) (BioDiagnostic Co.) spectrophotometrically.

### ELISA

α-Fetoprotein (AFP), β-catenin (Cat. Number. MBS700622 MyBioSource, Cat. Number. MBS843456 Mybiosource, respectively). E-cadherin, TGF-β (ab202413) Abcam, (ab119558) Abcam, respectively. Fascin (Cat. Number. MBS266620 Mybiosource) levels were analysed using commercially available ELISA kits.

### Quantitative real-time polymerase chain reaction (RT-PCR)

Real-Time-Polymerase Chain Reaction (RT-PCR) was used for determination of SMAD4 and α-SMA gene expression. Total RNA was isolated from liver tissues by using the RNeasy Purification Reagent (Qiagen, Valencia, CA, USA). The list of used primers for SMAD4 and α-SMA were showed in Table 1. Real time quantitative PCR was used to determine gene expression according to the instructions for Applied Bio systems version 3.1 software and SYBR Green I (Step One™, USA). β-actin gene was used as a housekeeping gene and an internal reference control and all data are expressed relative to it.

**Table 1.** Primer sets used in the PCR.

	Forward primer sequence	Reverse primer sequence
B-actin	5′-CAACCTTCTTGCAGCTCCTC −3′	5′-AGGGTCAGGATGCCTCTCTT −3′
α-SMA	5′-GTGACTACTGCCGAGCGTG-3′	5′-ATAGGTGGTTTCGTGGATGC-3′
SMAD4	5′-CATTCCTGTGGCTTCCACAA −3′	5′-GACTGATGGCTGGAGCTATT −3′

### Statistical analysis

The mean ± SEM was used to express data. Kolmogorov-Smirnov test was used to examine normality of sample distribution. Rat survival was assessed by using Kaplan–Meier method. One way ANOVA was used for determination of the differences among groups, followed by Bonferroni *post hoc* test. SPSS version 20 (IBM Corp.) was used to perform the statistical analysis. Statistical significance was predefined as *p* < 0.05.

## Results

### WRH-2412 effect on HCC-induced mortality and AFP elevation

**Figure 2.**
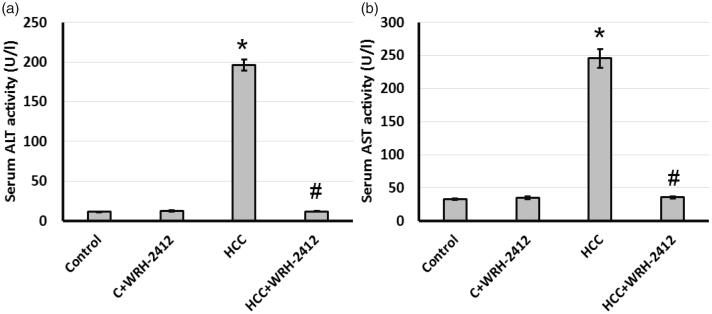
Effect of 5 mg/kg **WRH-2412** on survival rate and AFP serum levels in HCC rats. (A) Survival rate represented as Kaplan-Meier curve. (B) AFP serum levels in the experimental groups. Values are presented as the mean ± SEM, *p < 0.05 vs. control; ^#^*p* ≤ 0.05 vs. HCC group; AFP: α-fetoprotein; HCC: hepatocellular carcinoma; C: control.

The rat survival rate was increased from 30% in the HCC group to 90% in HCC rats treated with 5 mg/kg of **WRH-2412**. Control rats treated with **WRH-2412** at 5 mg/kg exhibited 100% survival at the end of the experiment. In addition, the percent of rats’ survival was associated with a significant reduction in the serum level of AFP compared with the control group (966 ng/mL in HCC group and 26.48 ng/mL in HCC rats treated with **WRH-2412**) (Figure 2).

### Effect of WRH-2412 on liver function tests

**Figure 3.**
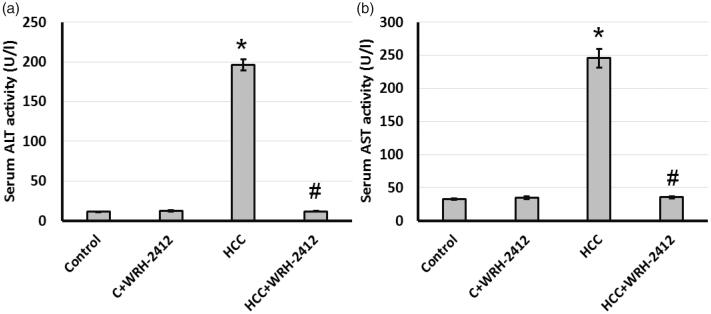
Effect of **WRH-2412** on serum liver markers levels in HCC rats. (a) ALT (b) AST levels. Values are expressed as the mean ± SEM, *p < 0.05 vs. control; ^#^*p* < 0.05 vs. HCC group; ALT: alanine aminotransferase; AST: aspartate aminotransferase; HCC: hepatocellular carcinoma; C: control.

Compared with the control group, serum ALT and AST activities were significantly elevated in the HCC group. Treating HCC rats with 5 mg/kg **WRH-2412** resulted in a significant reduction in serum ALT and AST activities compared with HCC group. These results suggested **WRH-2412** had hepatoprotective effects against HCC rats (Figure 3).

### Effect of WRH-2412 on HCC-induced morphological changes

**Figure 4.**
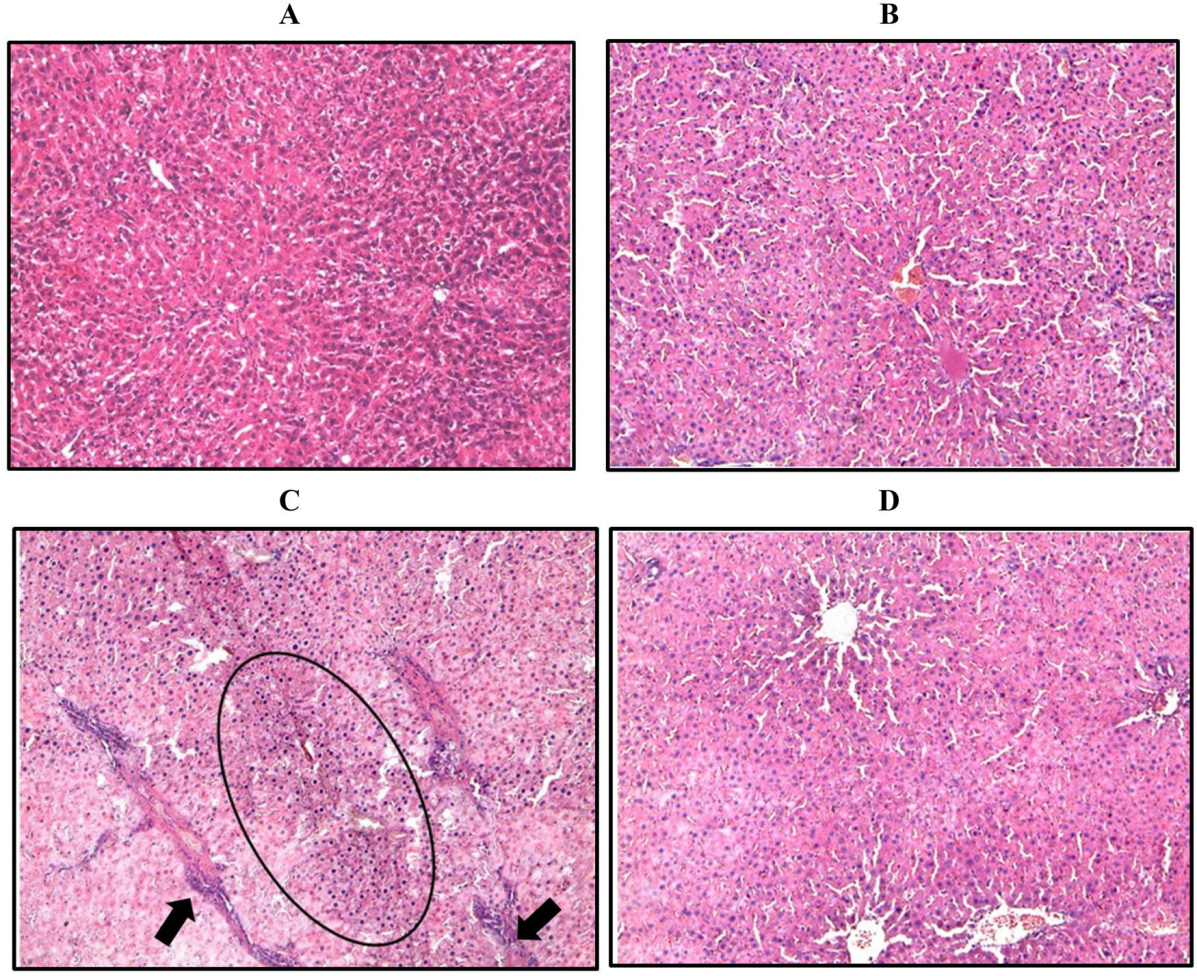
Representative image of hepatic sections stained with H/E. (A) Control group. (B) Control group treated with 5 mg/kg **WRH-2412**. (C) The liver architecture of HCC group showed massive break down of hepatic tissue together with hyperplastic nodules (Encircled) and apparent heteromorphism. (D) **WRH-2412** treated rats showed greatly reduction in these histopathological features in the liver.

Control group revealed normal appearance of hepatic lobule and liver architecture. While, the hepatocyte architecture in HCC group showed massive breakdown of hepatic tissues, with hyperplastic nodules (Encircled) and apparent heteromorphism. The nuclei were prominent and occupied most of the cells. Prominent connective tissue septa with many blood vessels are also noticed (arrow). The liver of rats treated with **WRH-2412** showed a significantly reduction in these histopathological features (Figure 4).

### Effect of WRH-2412 on HCC-induced expression of TGF-β

**Figure 5.**
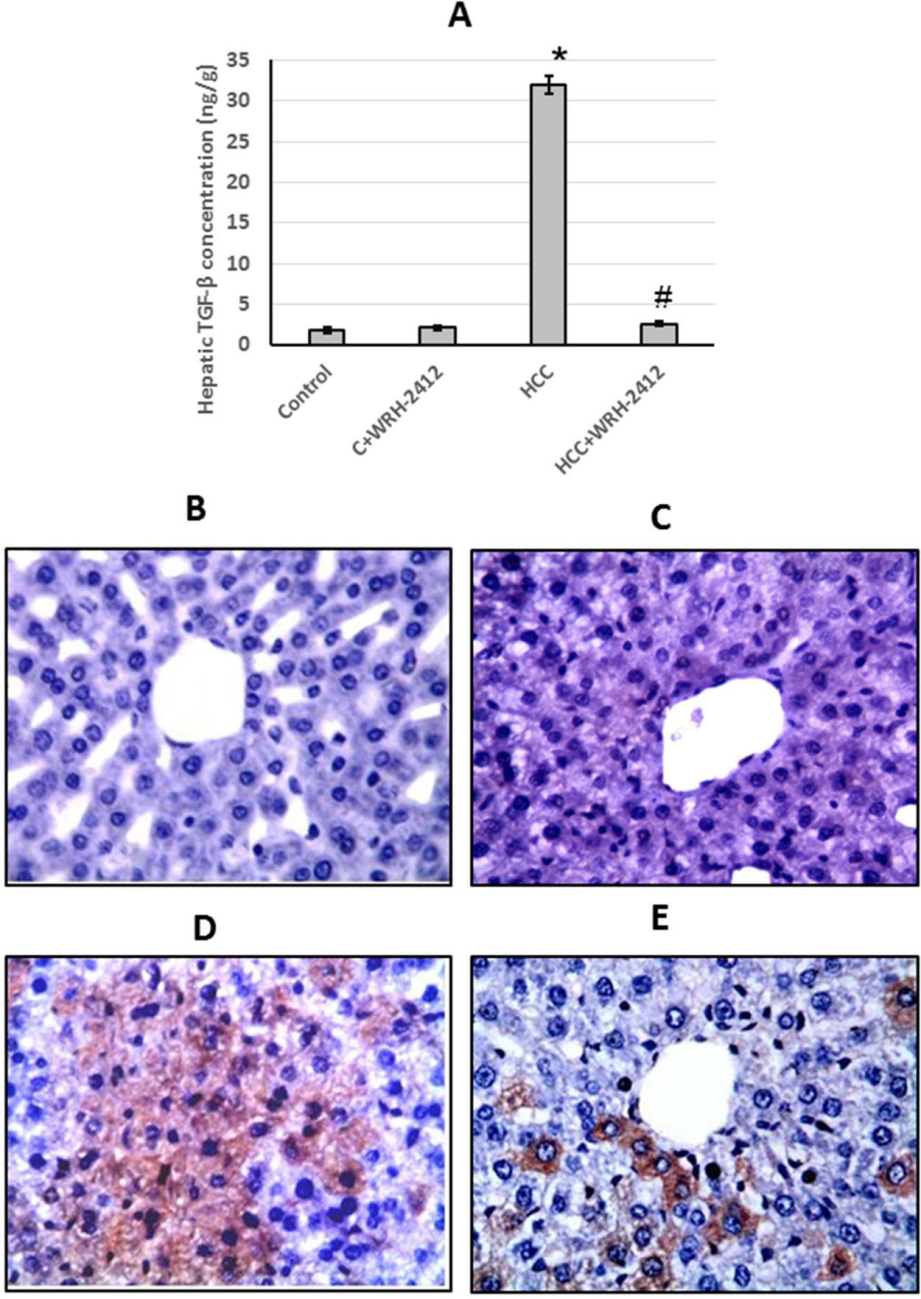
Effect of 5 mg/kg **WRH-2412** on TGF-β protein levels (A) as well as liver sections stained with anti- TGF-β antibody in control group (B), control group treated with **WRH-2412** (C), HCC group (D) and HCC group treated with **WRH-2412** (E). Values are expressed as the mean ± SEM, *p < 0.05 vs. control; ^#^*p* ≤ 0.05 vs. HCC group. TGF-β: transforming growth factor-β; HCC: hepatocellular carcinoma; C: control.

HCC rats showed a significant increase (17.29-fold) in TGF-β protein expression levels compared to the control groups. In addition, liver sections stained with anti-TGF-β antibodies showed increased immunostaining in sections from HCC group as compared to the control group. However, **WRH-2412** administration resulted in a significant decrease in TGF-β protein expression levels as compared with the HCC group with no effect on the control group (Figure 5).

### Effect of WRH-2412 on E-cadherin expression

**Figure 6.**
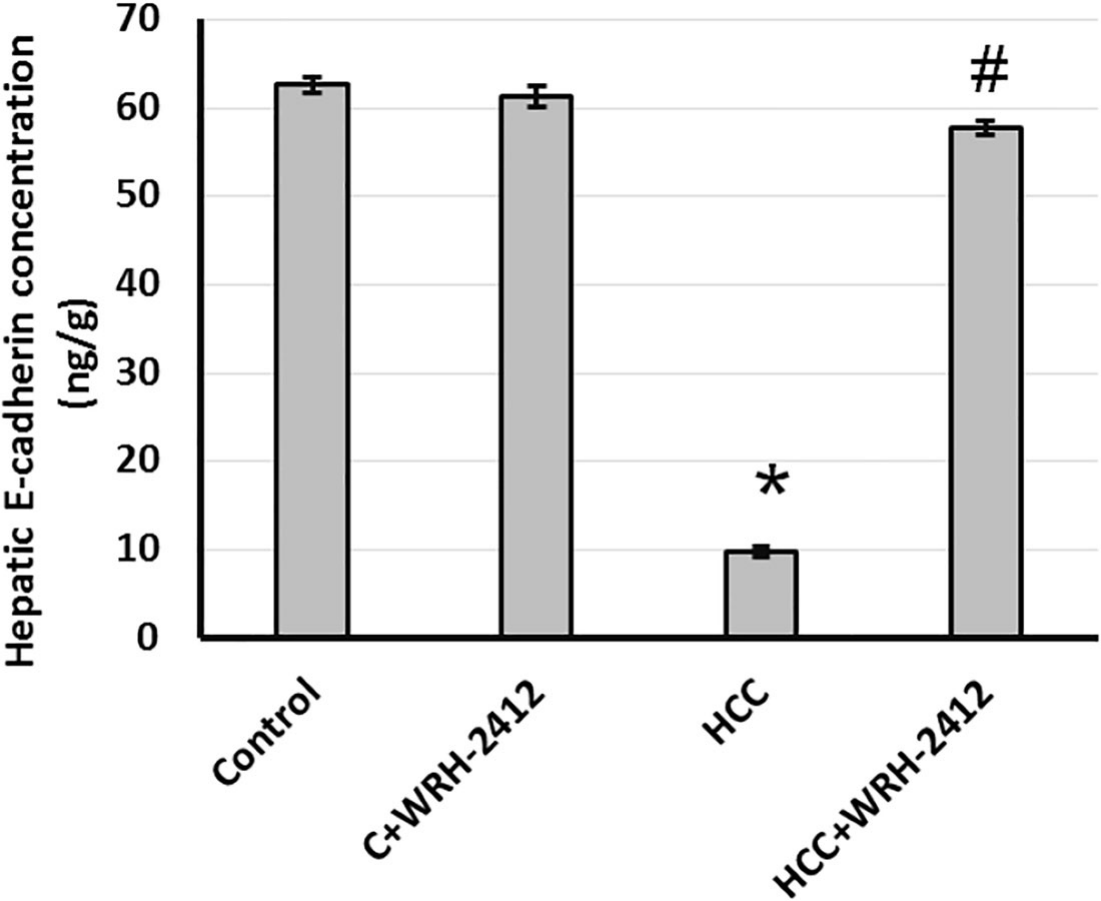
Effect of 5 mg/kg **WRH-2412** on hepatic protein level of E-cadherin. Values are expressed as the mean ± SEM, *p < 0.05 vs. control; ^#^*p* < 0.05 vs. HCC group; HCC: hepatocellular carcinoma; C; control.

HCC resulted with 83% reduction in E-cadherin expression level compared with the control groups. Nevertheless, HCC rats treated with **WRH-2412** resulted in reversion of this effect (Figure 6).

### Effect of WRH-2412 on β-catenin expression

**Figure 7.**
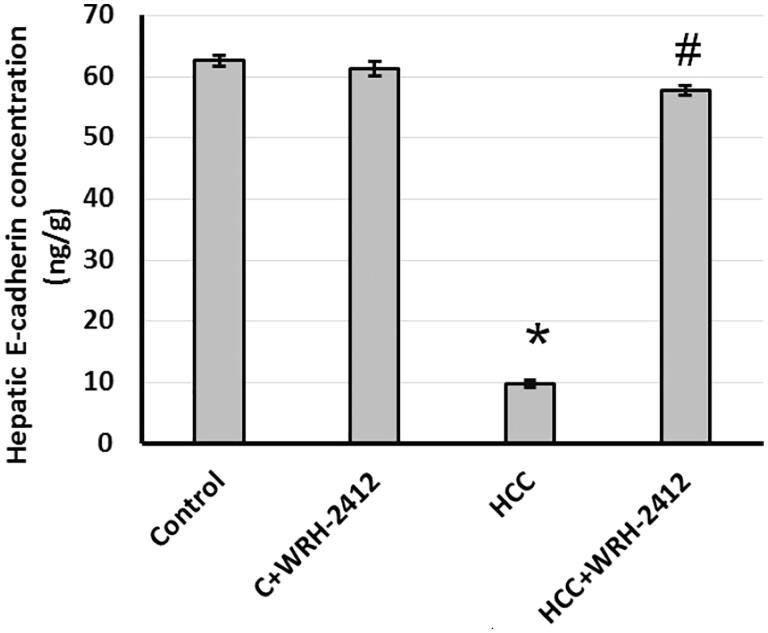
Effect of 5 mg/kg **WRH-2412** on hepatic protein level of E-cadherin. Values are expressed as the mean ± SEM, *p < 0.05 vs. control; ^#^*p* < 0.05 vs. HCC group; HCC: hepatocellular carcinoma; C: control.

**Figure 8.**
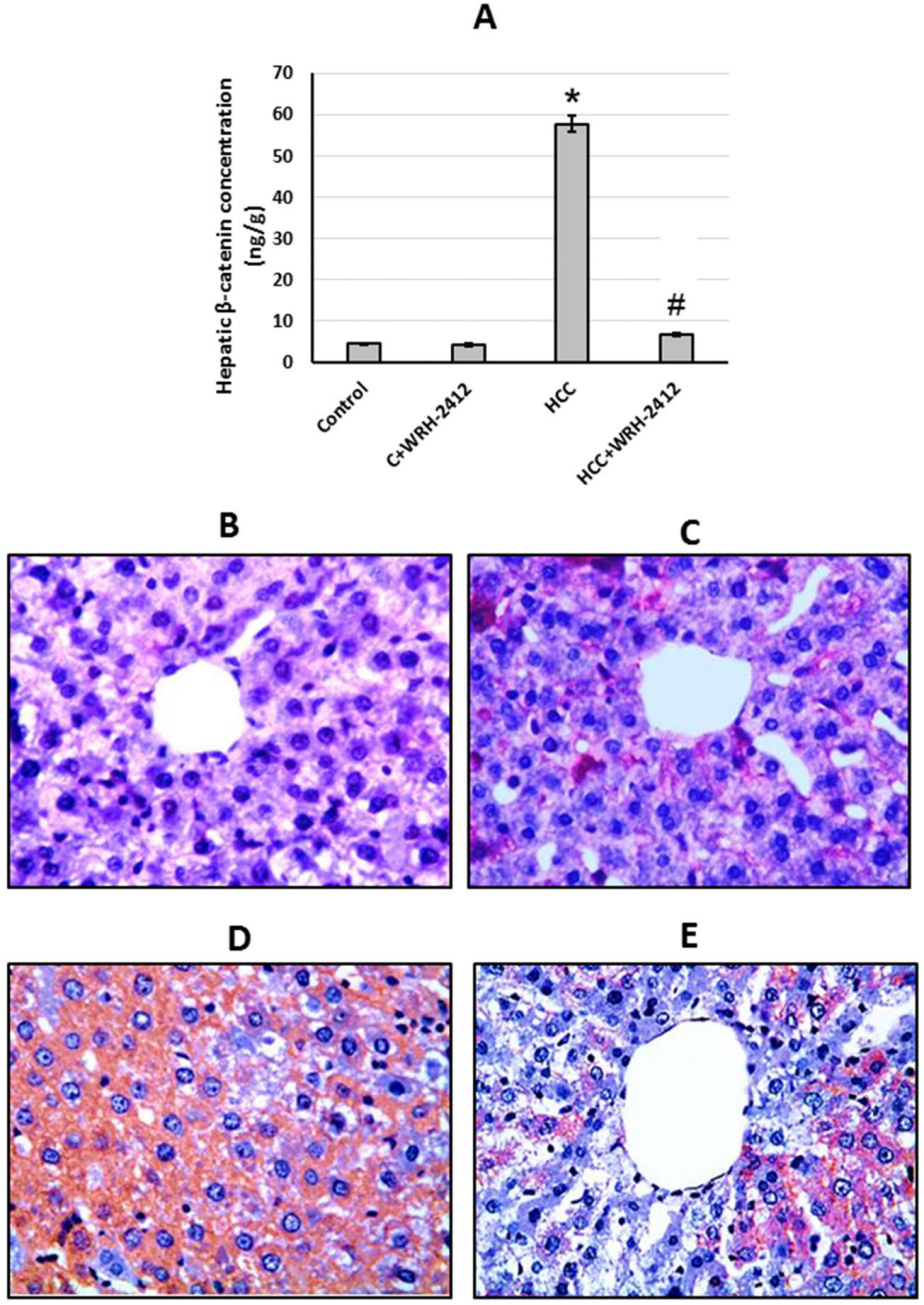
Effect of 5 mg/kg **WRH-2412** on β-catenin protein levels (A) as well as liver sections stained with anti- β-catenin antibody in control group (B), control group treated with **WRH-2412** (C), HCC group (D) and HCC group treated with **WRH-2412** (E). *p < 0.05 vs. control; ^#^*p* ≤ 0.05 vs. HCC group; HCC: hepatocellular carcinoma; C: control.

HCC resulted in elevation (12.95-fold) in β-catenin protein expression levels in comparison to the control groups. In addition, liver sections which stained with anti-β-catenin antibodies showed increased immunostaining in sections from HCC group as compared with the control group. Even so, HCC rats treated with **WRH-2412** resulted in significant reduction in the expression of β-catenin in HCC rats with no effect on the control rats (Figure 7 and 8).

### Effect of WRH-2412 on SMAD4 expression

**Figure 9.**
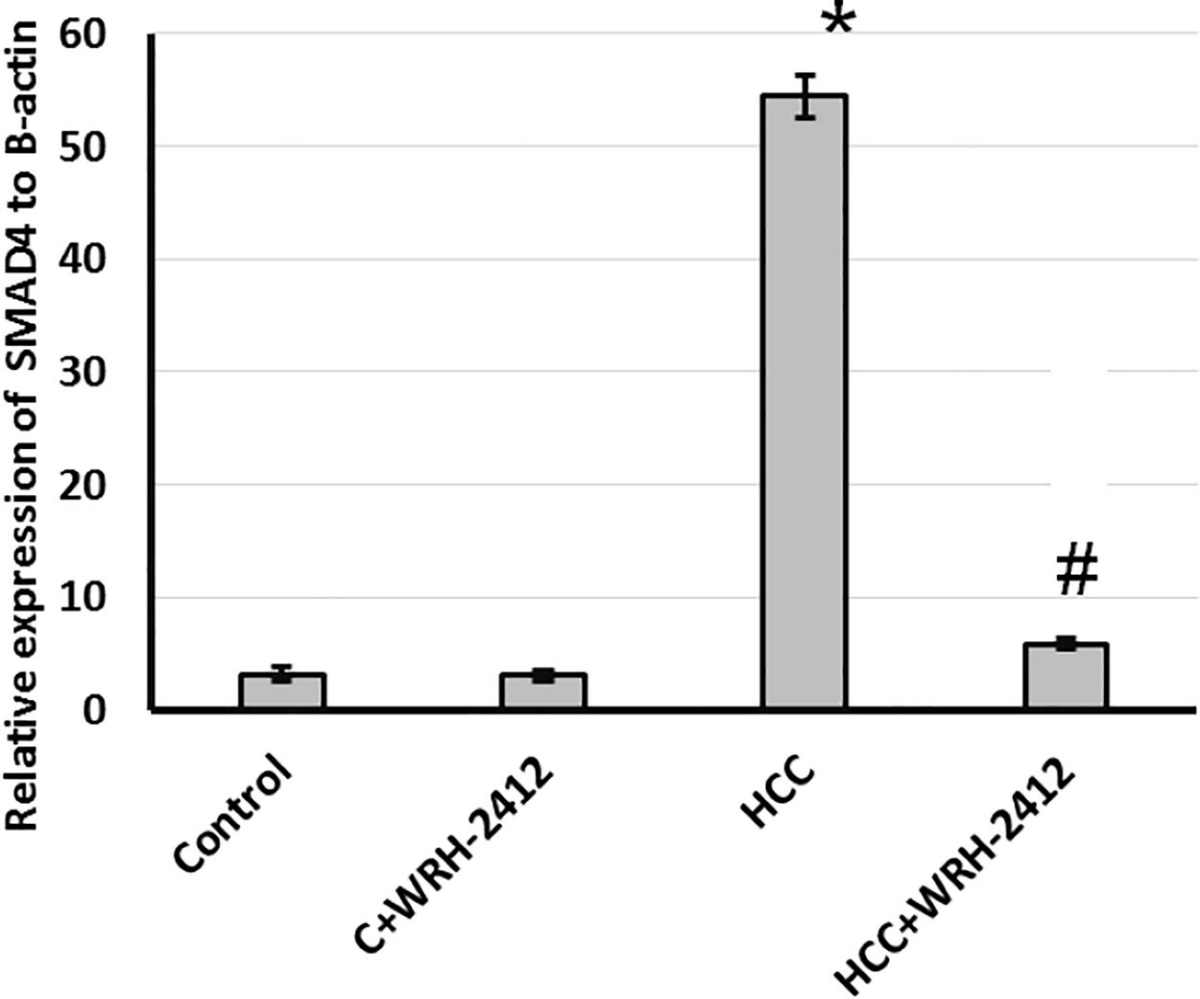
Effect of 5 mg/kg **WRH-2412** on hepatic protein level of SMAD4. Values are expressed as the mean ± SEM, *p < 0.05 vs. control; #*p* < 0.05 vs. HCC group; HCC: hepatocellular carcinoma; C: control.

Furthermore, evaluation of hepatic levels of SMAD4 showed significant increase in HCC rats as compared to the control rats. This elevation is blocked by treatment of rats with **WRH-2412** with no effect on the control group (Figure 9).

### Effect of WRH-2412 on vascular invasion markers

**Figure 10.**
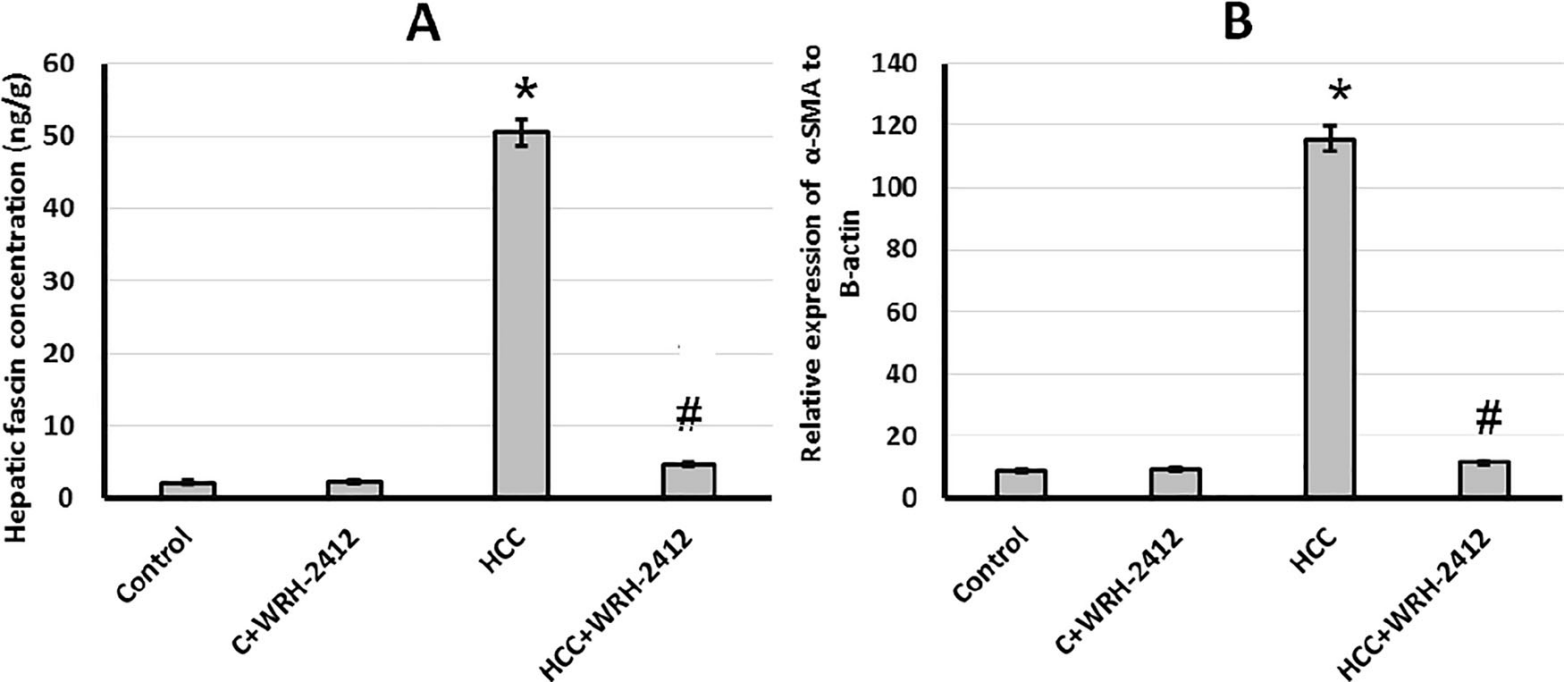
Effect of 5 mg/kg **WRH-2412** on vascular invasion markers. (A) Fascin and (B) *α*-SMA protein levels in the experimental groups. Values are expressed as the mean ± SEM, *p < 0.05 vs. control; #*p* < 0.05 vs. HCC group; HCC: hepatocellular carcinoma; C: control.

HCC rats demonstrated 22.58- and 12.95-fold increase in fascin protein expression and *α*-SMA gene expression levels, respectively, compared with the control groups. Nevertheless, HCC group treated with 5 mg/kg **WRH-2412** resulted in reduction of both fascin and *α*-SMA expression compared to HCC group with no effect on the control group (Figure 10).

## 4. Discussion

HCC is considered the sixth widespread cancer and the fourth worldwide cause of cancer-related death. There are three systemic drugs approved by FDA for advanced HCC treatment. The microenvironment inside the liver tissues contains many compounds such as cytokines, growth factors and extracellular matrix (ECM) as well as, many other cells as immune cells, kupffer cells, endothelial cells and fibroblasts. All these supplemented the hepatic carcinogenic tissue microenvironment with many valuable resources that help the development of *de-novo* HCC tumours.^12^

The first line therapy is represented by sorafenib and lenvatinib, while regorafenib is considered the second line of treatment.^13^ Although the most effective treatment of patients is liver resection and transplantation, these techniques are not suitable for most patients. Therefore, HCC is placed as the second most lethal cancer, with about five-year survival of 18%. One of the candidate therapeutic agent against HCC are the small molecules, pyrazolo[3,4-*b*]pyridine compounds, such as **WRH-2412** with many therapeutic activities as antiviral and anticancer. Treatment of HCC rats with **WRH-2412** proved promising antitumor activity. This can be proved by **WRH-2412** ability to reduce HCC-induced elevation in serum α-fetoprotein and number of nodules in the liver. Furthermore, the percent of HCC rats survival was doubled by using **WRH-2412**. In addition, **WRH-2412** produced many improvements in the structure of hepatic histopathological features as indicated by attenuation of destroyed cells, reduction of hyperplastic nodules and heteromorphism. It is the first time to prove the ability of **WRH-2412** to produce anticancer activity against experimentally induced HCC.

TGF-β is a pleiotropic, potent, regulatory cytokine and a major component that leads to EMT. Its signaling pathway has a great role in many cellular processes as cellular proliferation, differentiation, migration, apoptosis, adhesion, angiogenesis, immune surveillance and survival.^14^ In normal cases or in early stages of tumour TGF-β has tumour suppressor effects, while in advanced tumours it has a tumour progression and metastasis effects. Activation of TGF-β receptor with subsequent enhancement of β-catenin/SMAD4 pathway leads to many processes inside the tumour cells as cell reprogramming induction, epithelial phenotyping of primary tumour cells to acquire interstitial cell characteristics and tumour cell invasion to ECM leading to tumour metastasis.^15^ TGF- β has been recently considered as possible liver tumour marker.^16^ However, β-catenin is involved in regulating EMT and upregulated in tumours. β-catenin is a complicated protein that plays an important role in regulating several physiological processes, such as differentiation, proliferation, and tissue homeostasis and directly linked to HCC.^17^ It is associated with cell-to-cell adhesion and activation of the components of ECM. It is activated in about 30% of patients with HCC.^18^ Finally, one of the downstream of TGF-β/β-catenin is SMAD4, which is overexpressed in many types of cancer. In addition, deletion of SMAD4 gene produced protective effects against pancreatic cancer.^19^ However, we found that HCC results in overexpression of TGF-β, β-catenin and SMAD4 in rats. HCC rats treated with **WRH-2412** reversed all of these effects in HCC rats without affecting the control rats. It it the first time to report that **WRH-2412** protects against HCC through blocking TGF-β/β-catenin/SMAD4 axis.

Vascular invasion is present in about 25–50% of HCC patients and represents a great risk factor that enhances tumour recurrence and leads to poor overall survival among patients with HCC.^20^ Blood flowing into the HCC is done via neovascularization from arterial vessels and taken out primarily by via portal vein. The spread of HCC cells using the portal vein is considered as the major mechanism of intrahepatic metastasis.^21^ There is a cross talk between the HCC cells, microenvironment and ECM which might lead to vascular invasion.^22^ One of the vascular invasion markers is fascin, which is an actin-binding protein. Fascin regulates cell motility. Fascin is not expressed in normal cells, while it is overexpression during tumour invasion and metastasis.^23^ It helps in breaking intercellular junctions, enhancing cell movement and modifying ECM leading to tumour metastasis.^24^ To enhance tumour invasion, fascin needs the help of many other factors. In addition, *α*-SMA activation is linked to activation of myofibroblasts. It is associated with progression of several types of malignancy as osteosarcomas, lung adenocarcinoma, head and neck squamous cell carcinoma.^25^ Finally, E-cadherin is a transmembrane protein that plays a key role in establishing stable adherent junctions. It is associated with dedifferentiation, infiltration and metastasis in many cancers.^26^ We found that HCC results in increased expression of fascin and *α*-SMA associated with reduction in E-cadherin expression. Treatment of HCC rats with **WRH-2412** reversed these effects in HCC rats without affecting the control rats.

## Conclusion

The present study indicated that **WRH-2412** has an antitumor effects and may suppress HCC development *via* inhibition of the TGF-β/β-catenin/*α*-SMA pathway axis, which may suppress vascular invasion markers. Therefore, **WRH-2412** stands out as a promising starting point and as a novel potential therapeutic drug for improving the outcome of patients with HCC.
