# Correction

**DOI:** 10.1080/14756366.2023.2218746

**Published:** 2023-06-05

**Article title:** WRH-2412 alleviates the progression of hepatocellular carcinoma through regulation of TGF-β/β-catenin/α-SMA pathway

**Authors:** Mohammed A. F. Elewa, Wagdy M. Eldehna, Ahmed M. E. Hamdan, Samraa H. Abd El-kawi, Asmaa M. El-Kalaawy, Taghreed Majrashi, Reham F. Barghash, Hatem A. Abdel-Azizb, Khalid S. Hashem, Mohammed M. Al-Gayyar

**Journal:** *Journal of Enzyme Inhibition and Medicinal Chemistry*

**Bibliometrics:** Volume 38, Number 1

**DOI:** https://doi.org/10.1080/14756366.2023.2185761

When the article has been previously published, an incorrect version of the figures were included. The correct version of the figures is as follows:

**Figure 1.**
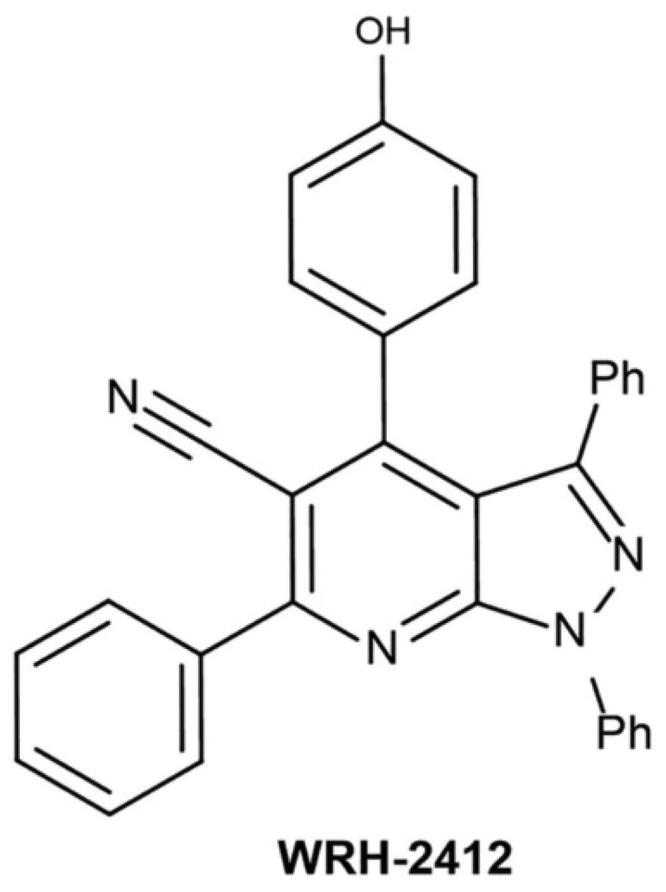
Chemical structure of **WRH-2412**.

**Figure 2.**
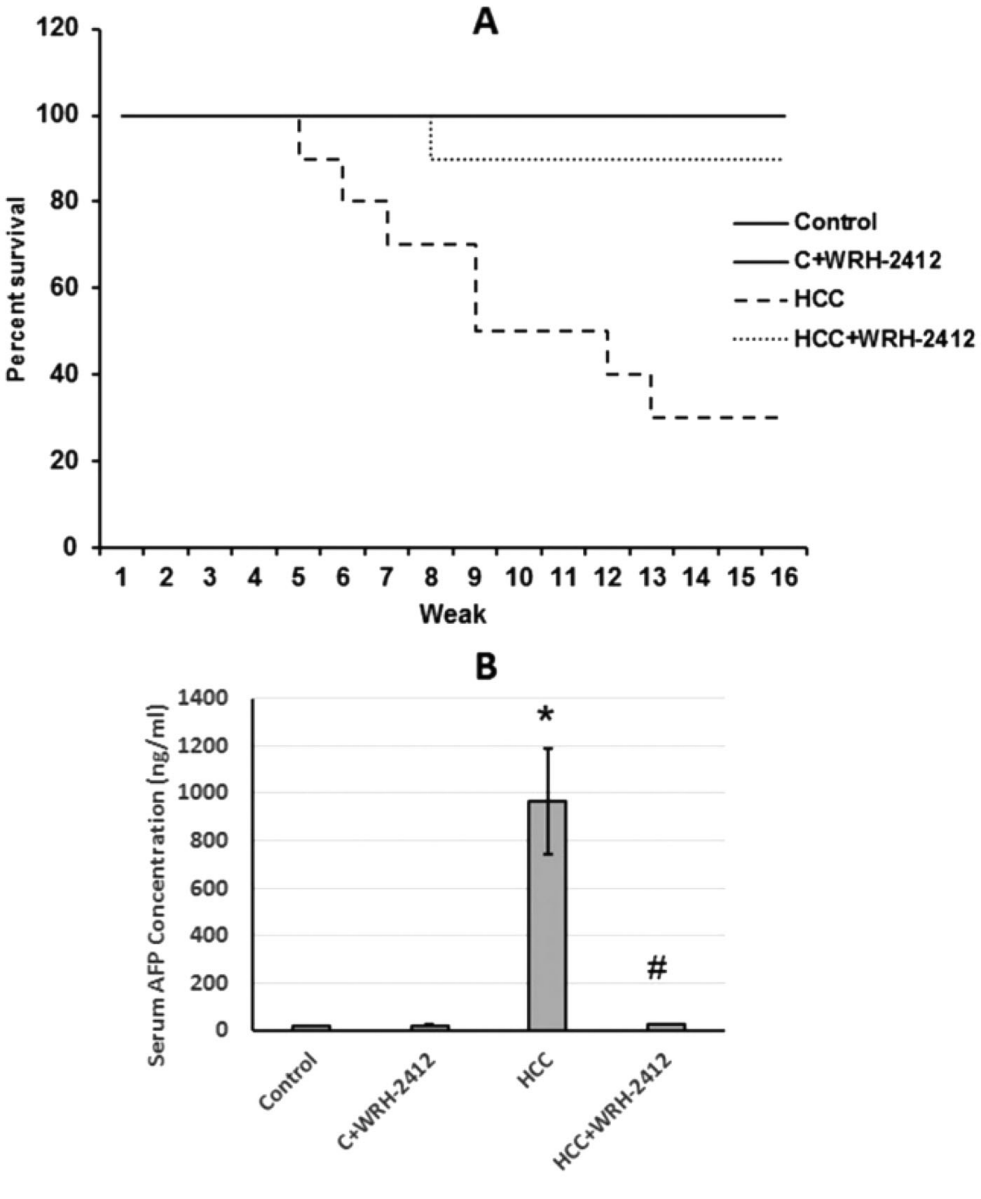
Effect of 5 mg/kg **WRH-2412** on survival rate and AFP serum levels in HCC rats. (A) Survival rate represented as Kaplan-Meier curve. (B) AFP serum levels in the experimental groups. Values are presented as the mean ± SEM, *p < 0.05 vs. control; ^#^*p* ≤ 0.05 vs. HCC group; AFP: α-fetoprotein; HCC: hepatocellular carcinoma; C: control.

**Figure 3.**
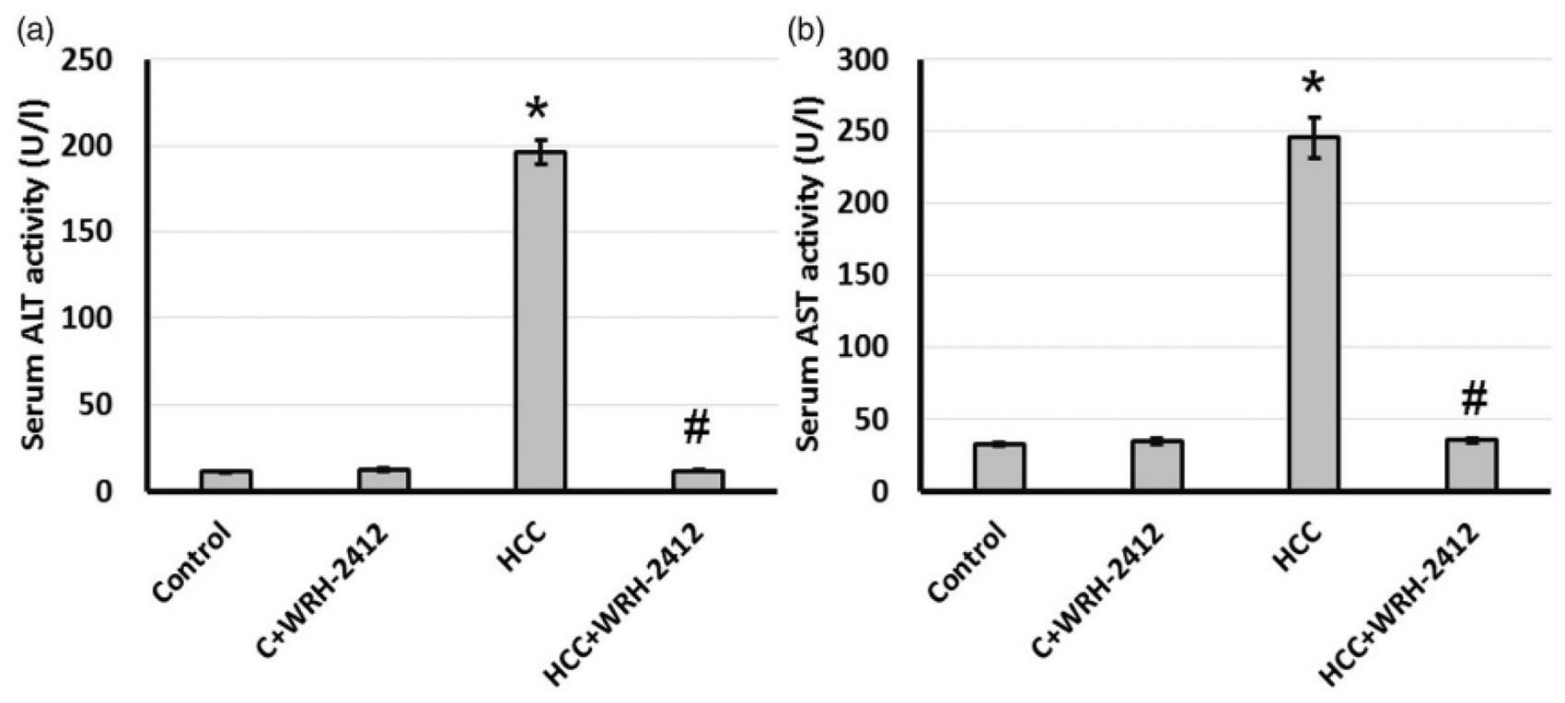
Effect of **WRH-2412** on serum liver markers levels in HCC rats. (a) ALT (b) AST levels. Values are expressed as the mean ± SEM, *p < 0.05 vs. control; ^#^*p* < 0.05 vs. HCC group; ALT: alanine aminotransferase; AST: aspartate aminotransferase; HCC: hepatocellular carcinoma; C: control.

**Figure 4.**
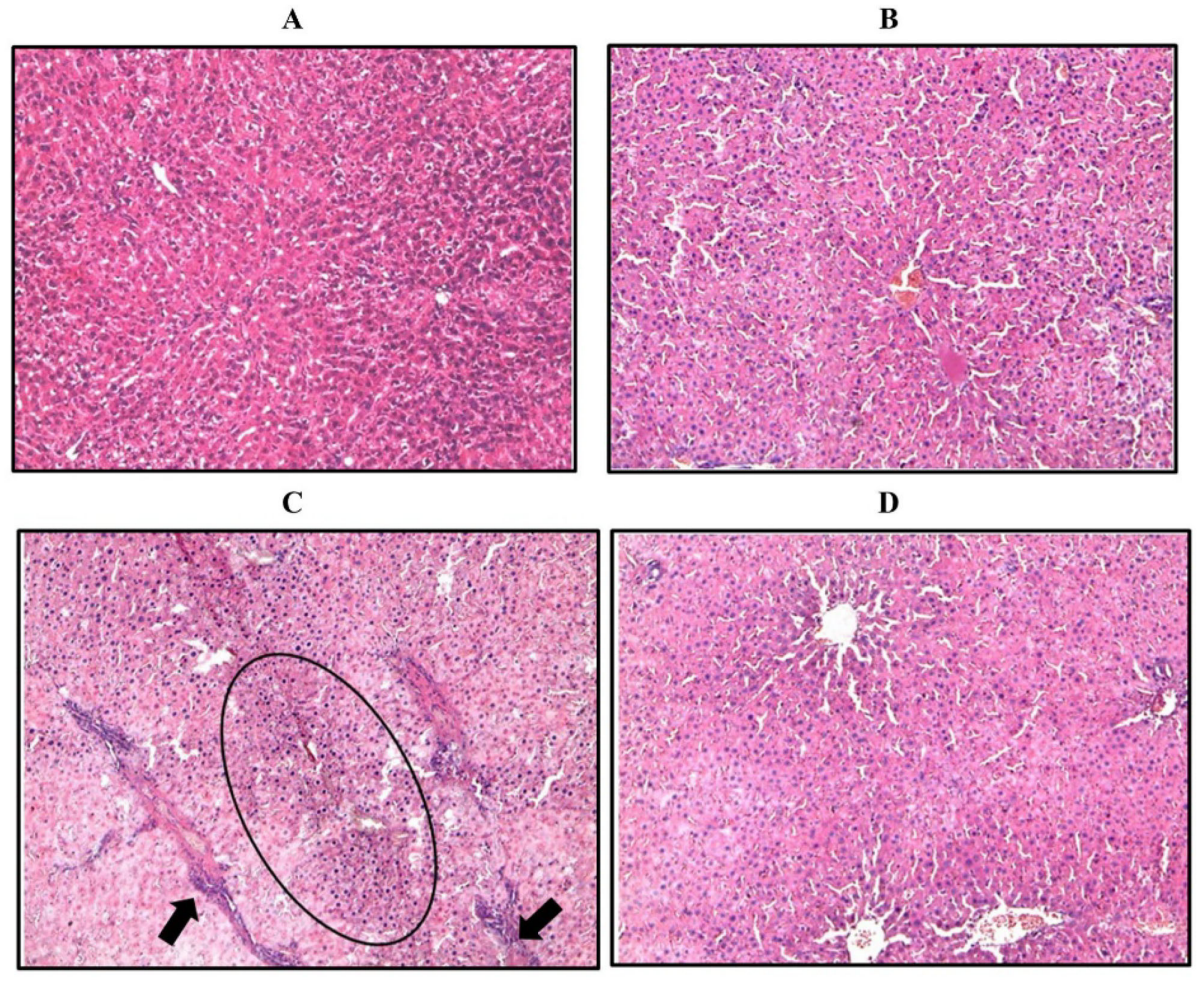
Representative image of hepatic sections stained with H/E. (A) Control group. (B) Control group treated with 5 mg/kg **WRH-2412**. (C) The liver architecture of HCC group showed massive break down of hepatic tissue together with hyperplastic nodules (Encircled) and apparent heteromorphism. (D) **WRH-2412** treated rats showed greatly reduction in these histopathological features in the liver.

**Figure 5.**
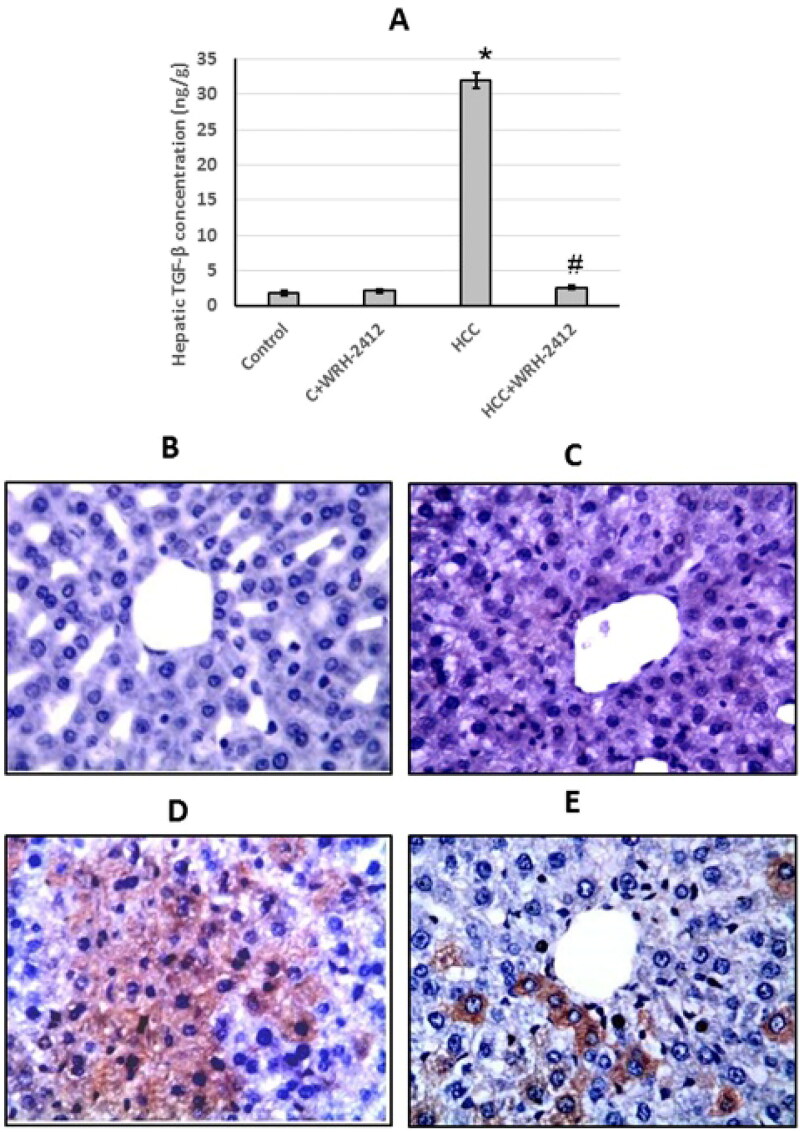
Effect of 5 mg/kg **WRH-2412** on TGF-β protein levels (A) as well as liver sections stained with anti- TGF-β antibody in control group (B), control group treated with **WRH-2412** (C), HCC group (D) and HCC group treated with **WRH-2412** (E). Values are expressed as the mean ± SEM, *p < 0.05 vs. control; ^#^*p* ≤ 0.05 vs. HCC group. TGF-β: transforming growth factor-β; HCC: hepatocellular carcinoma; C: control.

**Figure 6.**
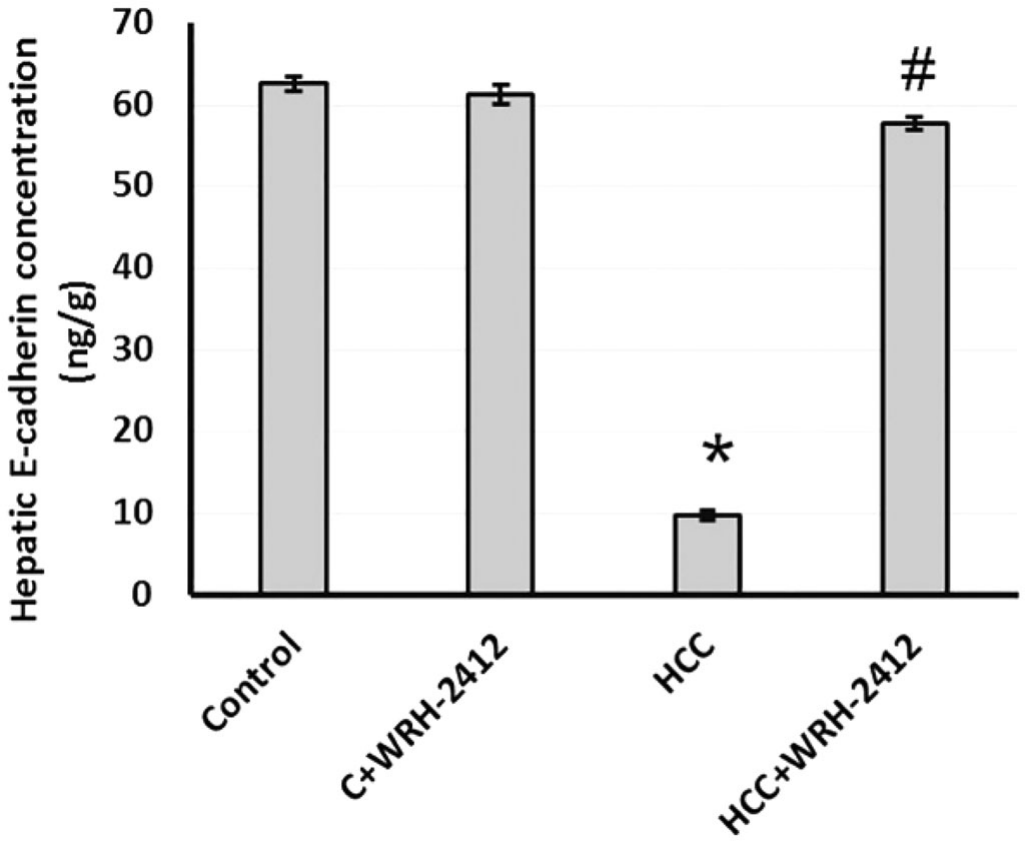
Effect of 5 mg/kg **WRH-2412** on hepatic protein level of E-cadherin. Values are expressed as the mean ± SEM, *p < 0.05 vs. control; ^#^*p* < 0.05 vs. HCC group; HCC: hepatocellular carcinoma; C; control.

**Figure 7.**
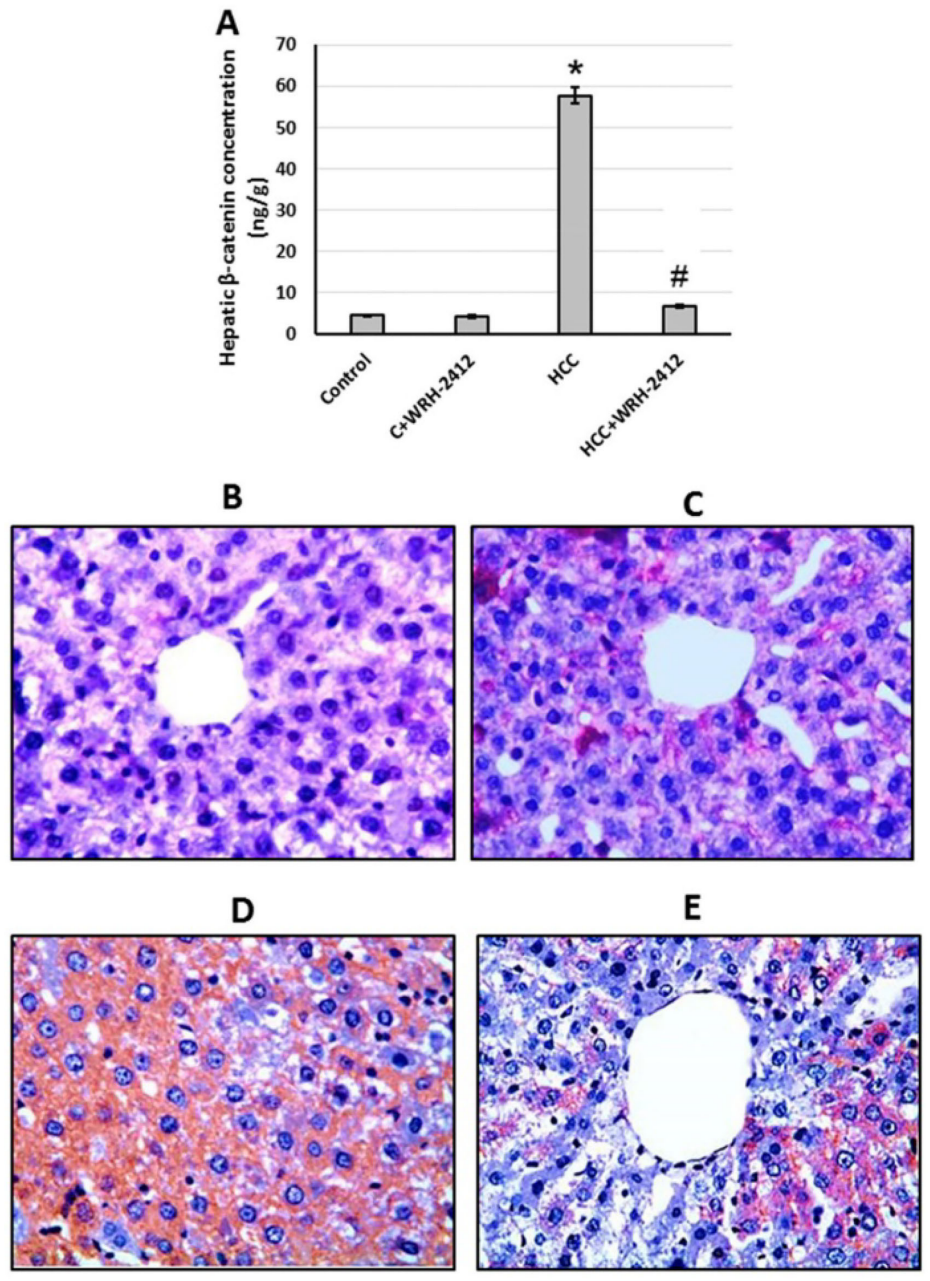
Effect of 5 mg/kg **WRH-2412** on β-catenin protein levels (A) as well as liver sections stained with anti- β-catenin antibody in control group (B), control group treated with **WRH-2412** (C), HCC group (D) and HCC group treated with **WRH-2412** (E). *p < 0.05 vs. control; ^#^*p* ≤ 0.05 vs. HCC group; HCC: hepatocellular carcinoma; C: control.

**Figure 8.**
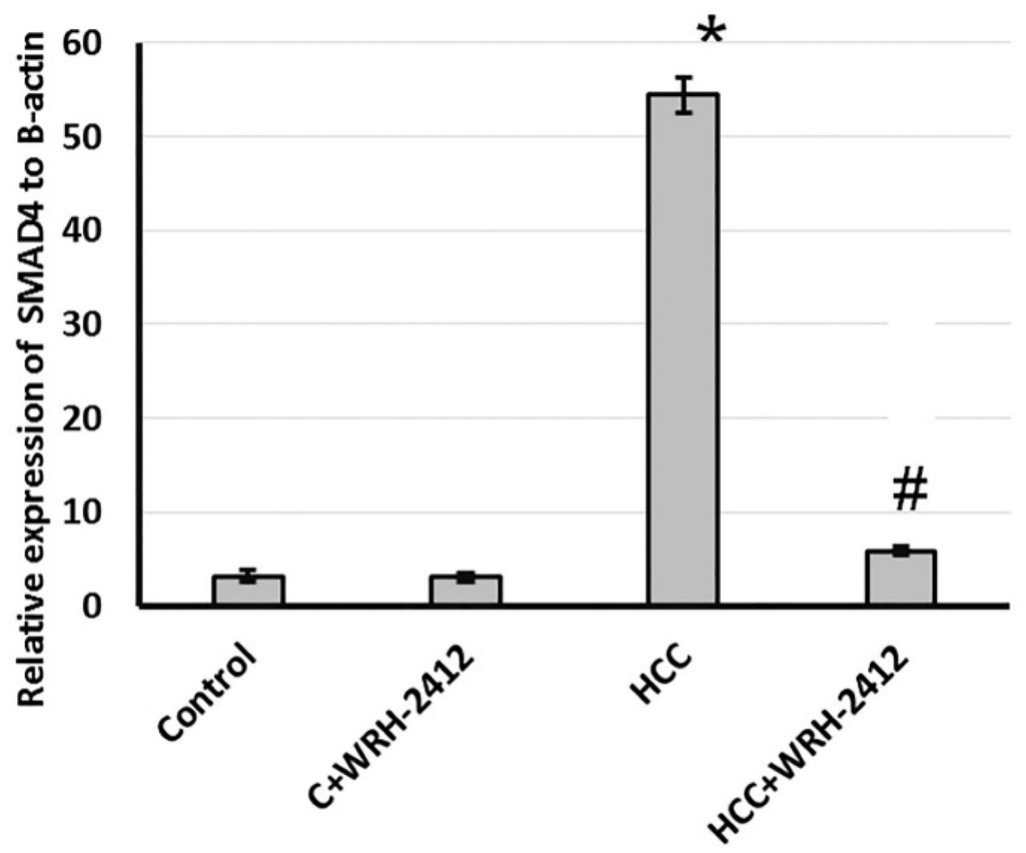
Effect of 5 mg/kg **WRH-2412** on hepatic protein level of SMAD4. Values are expressed as the mean ± SEM, *p < 0.05 vs. control; #*p* < 0.05 vs. HCC group; HCC: hepatocellular carcinoma; C: control.

**Figure 9.**
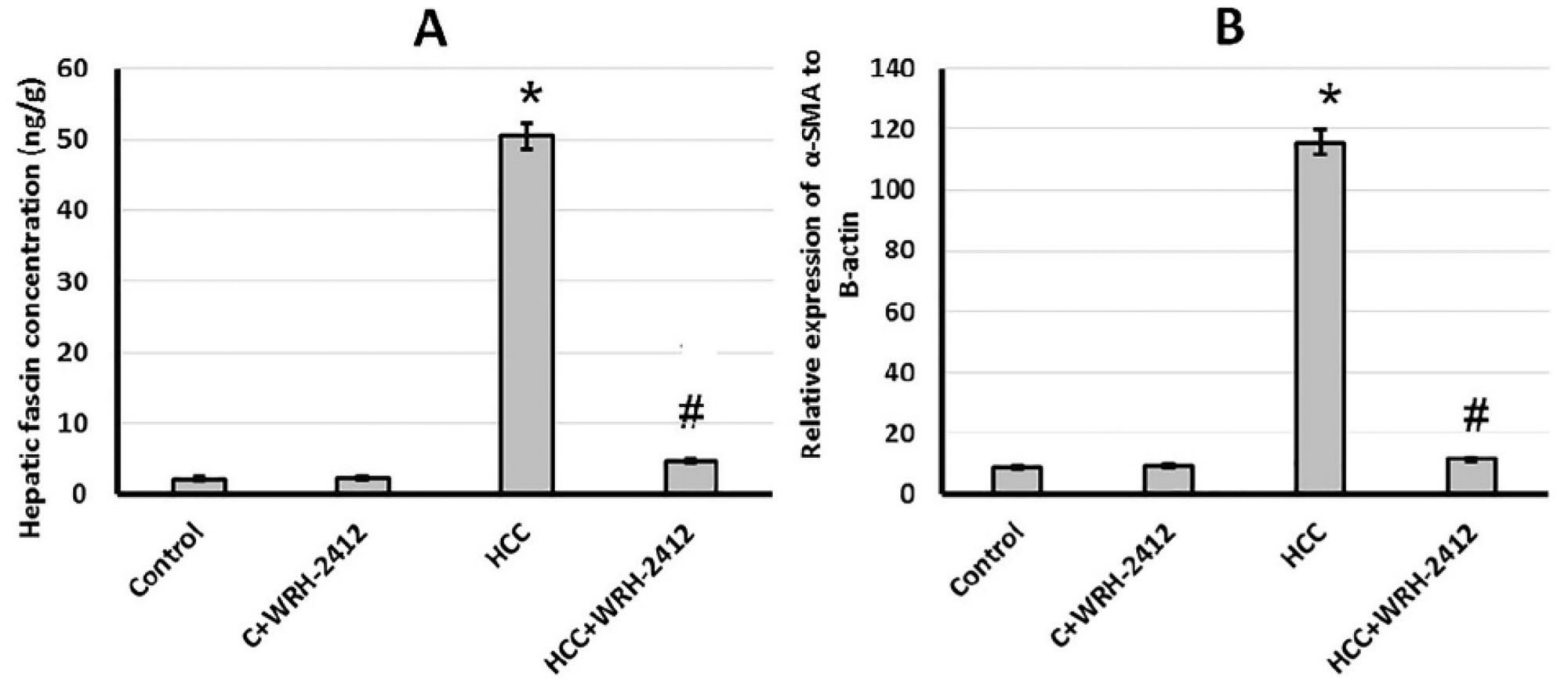
Effect of 5 mg/kg **WRH-2412** on vascular invasion markers. (A) Fascin and (B) *α*-SMA protein levels in the experimental groups. Values are expressed as the mean ± SEM, *p < 0.05 vs. control; #*p* < 0.05 vs. HCC group; HCC: hepatocellular carcinoma; C: control.

The authors are not responsible for the errors and the online version of this paper has been corrected.

